# Reconstruction with Modular Hemipelvic Endoprosthesis after Pelvic Tumor Resection: A Report of 50 Consecutive Cases

**DOI:** 10.1371/journal.pone.0127263

**Published:** 2015-05-26

**Authors:** Bo Wang, Xianbiao Xie, Junqiang Yin, Changye Zou, Jin Wang, Gang Huang, Yongqian Wang, Jingnan Shen

**Affiliations:** Department of Musculoskeletal Oncology, the First Affiliated Hospital of Sun Yat-Sen University, Guangzhou, Guangdong, China; Cardiff University, UNITED KINGDOM

## Abstract

**Purpose:**

To evaluate the effectiveness of reconstruction with a modular hemipelvic endoprosthesis after pelvic tumor resection.

**Methods:**

We retrospectively studied 50 consecutive patients diagnosed with pelvic tumor from 2003 to 2013. All patients received limb-salvage surgery and reconstruction with modular hemipelvic endoprosthesis.

**Results:**

Patients were followed for an average of 54 months. At the most recent follow-up, 32 patients were alive with an estimated three-year and five-year survival rate of 66.3% and 57.5% according to the Kaplan-Meier survival analysis. Eighteen patients died from the tumor, with a mean survival of 28 months, and 9 patients experienced local recurrence at an average of 19.6 months after surgery. Patients with marginal or intracapsular surgical margins had a significantly higher recurrence rate than those with wide margins (p=0.02). Metastasis occurred in 12 cases at an average of 16 months after surgery. The perioperative complication rate was 48.0%, and the most common complications were wound healing disturbance (28.0%) and deep infection (14.0%). The endoprosthetic complication rate was 16.0%, and breakage of the pubic connection plate was the most common complication. The mean Musculoskeletal Tumor Society score was 61.4%.

**Conclusion:**

Reconstruction with a modular hemipelvic endoprosthesis after pelvic tumor resection can improve function, with an acceptable complication rate.

## Introduction

Pelvic tumors, particularly malignant tumors, greatly impact patients’ survival and quality of life. The reconstruction of pelvic bone defects in orthopedic oncology remains challenging, particularly when the hemipelvis is considerably involved. Limb-salvage surgery with endoprosthetic replacement or biological reconstruction is favored by patients over classic hemipelvectomy[1–4]. Biological reconstruction, such as autografts, allografts, iliofemoral and ischiofemoral arthrodesis, are limited by extended bone and soft tissue defects and result in high complication rates and poor limb function[5–7]. Hemipelvic endoprosthetic reconstruction permits early ambulation and provides better short term function. However, the variety of prosthetic designs, combined with insufficient follow-up data and high complication rates makes hemipelvic endoprosthetic reconstruction controversial.

In this article, we report on 50 consecutive patients with widely invasive pelvic tumors who underwent surgical resection and reconstruction with a modular hemipelvic endoprosthesis at our center over the past 10 years. We evaluated the clinical outcomes and effectiveness of hemipelvic endoprosthetic reconstruction.

## Materials and Methods

### Patient Demographics

Fifty consecutive patients (29 males and 21 females; average age, 26 years; range, 12 to 67 years) underwent reconstructive surgery with a modular hemipelvic endoprosthesis after tumor resection at the Musculoskeletal Oncology Center of the First Affiliated Hospital of Sun Yat-Sen University between 2003 and 2013.Written informed consents were obtained from all patients or parents for those under 18 years old. The patient described in figures has given written informed consent (as outlined in PLOS consent form) to publish these case details. This study was approved by ethic committee of the First Affiliated Hospital of Sun Yat-Sen University.

Pathological diagnoses were confirmed via preoperative core needle biopsy and included 21 cases of osteosarcomas, 11 Ewing’s sarcomas, 8 chondrosarcomas, 5 metastatic malignancies, 3 fibrosarcomas and 2 giant cell tumors of bone (Table 1). All primary tumors were Enneking stage IIB[8]. The initial tumor location and type of surgical resection was assigned according to the Enneking and Dunham classification[9].

**Table 1:** A list of the pathological diagnoses.

Pathological diagnosis	No.
**Osteosarcoma**	21
**Ewing’s sarcoma**	11
**Chondrosarcoma**	8
**Metastasis**	5
**Fibrosarcoma**	3
**Giant cell tumor of bone**	2
**Total**	50

### Adjuvant Therapy

Neo-adjuvant chemotherapy was administered if indicated, according to the guidelines of the National Comprehensive Cancer Network (NCCN). Osteosarcoma treatment regimens included doxorubicin, cisplatin, methotrexate and ifosfamide. Ewing sarcoma was treated with vincristine, doxorubicin and ifosfamide. Except in 6 patients with conventional chondrosarcoma who did not receive chemotherapy, 37 patients with a primary malignant tumor received 2 cycles of chemotherapy preoperatively and 4–6 cycles postoperatively. Four patients with Ewing sarcoma underwent radiotherapy.

Good response to neo-adjuvant chemotherapy was assessed in terms of (1) relief of pain, (2) hardening or diminution of local mass, (3) decreased serum alkaline phosphatase (ALP) level for osteosarcoma, and (4) decreased mass volume or clearer tumor border with less soft tissue edema, according to MR scan.

### Surgery

#### Indications and Contraindications

Indications for surgery included (1) confirmed malignant or invasive pelvic tumor without metastasis beyond control, (2) good response to neo-adjuvant chemotherapy, (3) favorable surgical margin under limb salvage, and (4) no obvious invasion of the iliac vessels, sciatic nerve or femoral nerve.

Contraindications for surgery included (1) extensive invasion with poor response to chemotherapy, (2) nonstandard open biopsy leading to local tumor contamination, and (3) intolerance to surgical procedures due to poor general conditions.

#### Surgical Margin

Surgical margin was classified according to the system of Musculoskeletal Tumor Society (MSTS)[10]. Wide margins were required for primary tumors, while marginal and intralesional resections were both acceptable for metastatic malignancies.

#### Endoprosthesis

**Fig 1:**
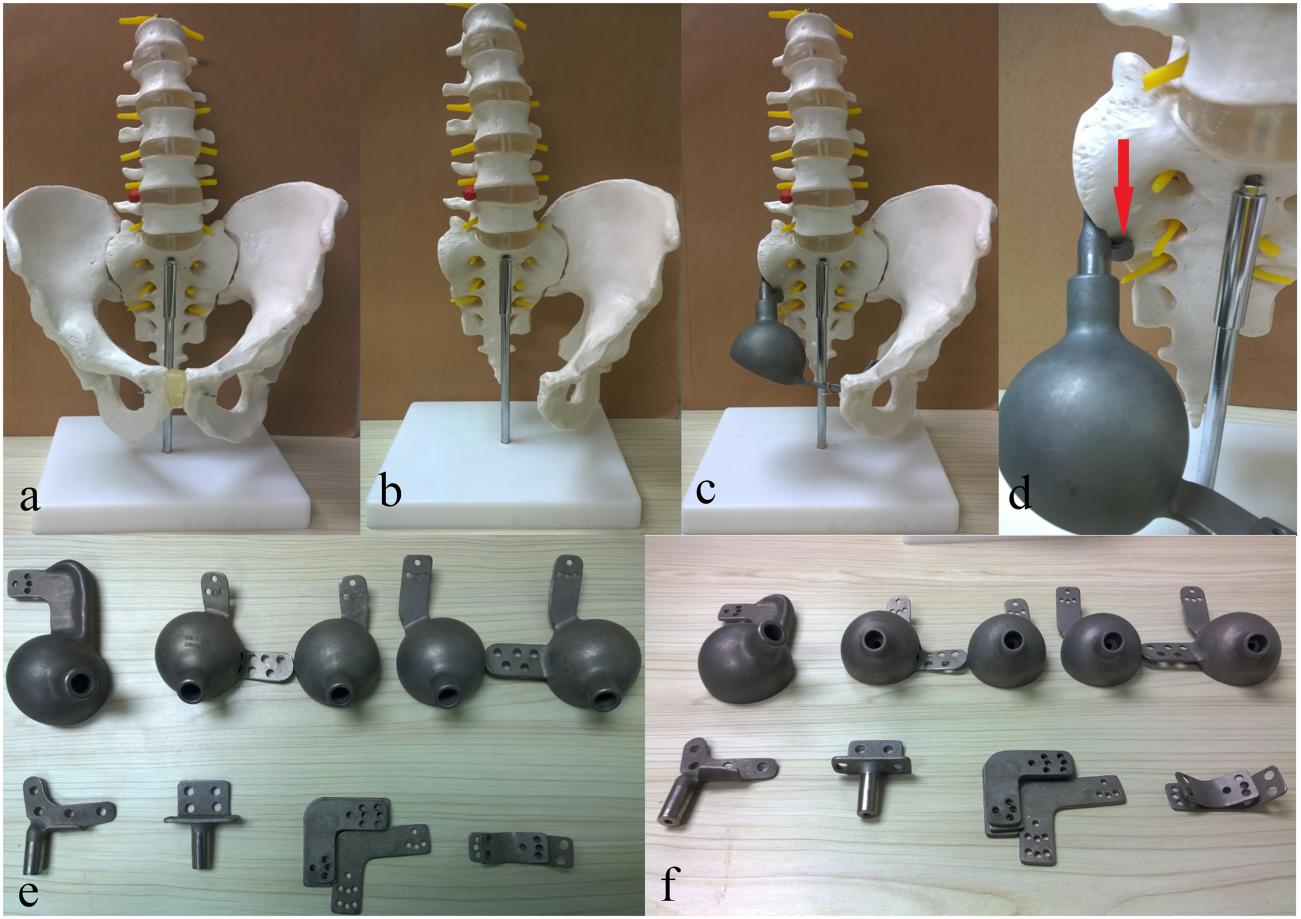
The modular hemipelvic endoprosthesis used in our study. (a) Preoperative model of pelvis. (b) Postoperative model after resection of tumor in which osteotomy was performed to sacroiliac joint and pubic symphysis. (c) Postoperative model reconstructed with modular hemipelvic endoprosthesis. (d) The sacral hook (indicated by a red arrow) transforming tearing forces of the sacral connecting screws into compressing stress. (e) and (f) The disassembled components of our modular hemipelvic endoprosthesis.

Reconstruction was completed with a modular hemipelvic endoprosthesis. The system was designed by Professor Shen Jingnan and manufactured by Lidakang Science and Technology Co. Ltd. Beijing, China. The prosthesis was composed of one restrained tumor total hip joint, one pubic connection plate, one sacral connection part and a sacral hook. The sacral hook was designed to transform high shearing force of the sacral fixation screws into compression stress while inserted and installed to the dome shaped anterior side of sacrum. Iliac wing was not anatomically reconstructed to allow a better soft tissue coverage and to reduce wound-related complications. Vancomycin-containing bone cement was used to seal and reinforce the prosthesis (Fig 1).

#### Surgical Procedure

The surgical procedures were identical to those previously reported[1]. A combination of an extended ilioinguinal and Smith-Petersen approach was used. The gluteus medius and minimus were often sacrificed to achieve wide surgical margins. The ipsilateral internal iliac artery was ligated for intraoperative hemorrhage control when necessary. Microwave ablation was employed to prevent tumor contamination and to reduce hemorrhage. The rotation center of the hip was usually shifted superomedially to reduce dead space and improve soft tissue coverage.

#### Postoperative Management

Second-generation cephalosporin was intravenously administered for infection prophylaxis for the first postoperative week and orally for another two weeks thereafter. Anti-thrombotic stockings and calf compression devices were routinely used for prophylaxis against deep vein thrombosis. Anticoagulants were administered when indicated. The hip joint was restricted to mild abduction and external rotation to prevent early dislocation. After 8 to 10 weeks of bed rest for scar tissue formation and construction stabilization, increased weight bearing was allowed, with the aid of crutches.

### Follow-up

Regular follow-up was scheduled every 3 months in the first 2 postoperative years, every 6 months for the following 3 years, and yearly thereafter. Physical examination, functional assessment, local radiographs and chest computed tomography (CT) scans were included in the follow-up evaluations. Function assessment was based on the MSTS scale[11]. It consisted of six factors such as pain, functional activities, emotional acceptance, external supports, walking ability, and gait. Each item was rated in a range of 0 to 5 (0 = bad function and 5 = normal function) with a maximum score of 30. The final score was usually converted to a percentage. Limb function was assessed at the latest follow-up.

### Statistical Analysis

Kaplan-Meier survival analysis was performed to estimate overall survival, local recurrence and distal metastasis, in which the events were defined as death, confirmed recurrence and metastasis, respectively. Surviving patients were censored at the last date of follow-up in the analysis of overall survival. Patients, dead or alive, without recurrence and metastasis were censored in the analysis of recurrence rate and metastasis rate. In addition, Kaplan-Meier survival analysis was used to evaluate survival rates of the prosthesis in which the event was defined as prosthetic revision for any reason. Log rank test was used to compare survival and recurrence rates between primary and metastatic tumors and recurrence rates between tumors with wide surgical margins and those with marginal or intracapsular margins. A p-value of less than 0.05 was considered to be significant. All statistical analyses were performed using the Statistical Package for the Social Science (SPSS) software, version 19.0 (SPSS Inc, Chicago, IL, USA).

## Results

Fifty patients were followed for an average of 54 months (range, 12–113 months). The resection types and surgical margins are described in Table 2.

**Table 2:** Types of resection and surgical margins.

Type of resection	No.	Wide resection	Marginal resection	Intralesional resection
**I+II**	21	16	3	2
**II+III**	12	5	4	3
**I+II+III**	17	8	5	4
**Total**	50	29	12	9

### Oncological Outcomes

**Fig 2:**
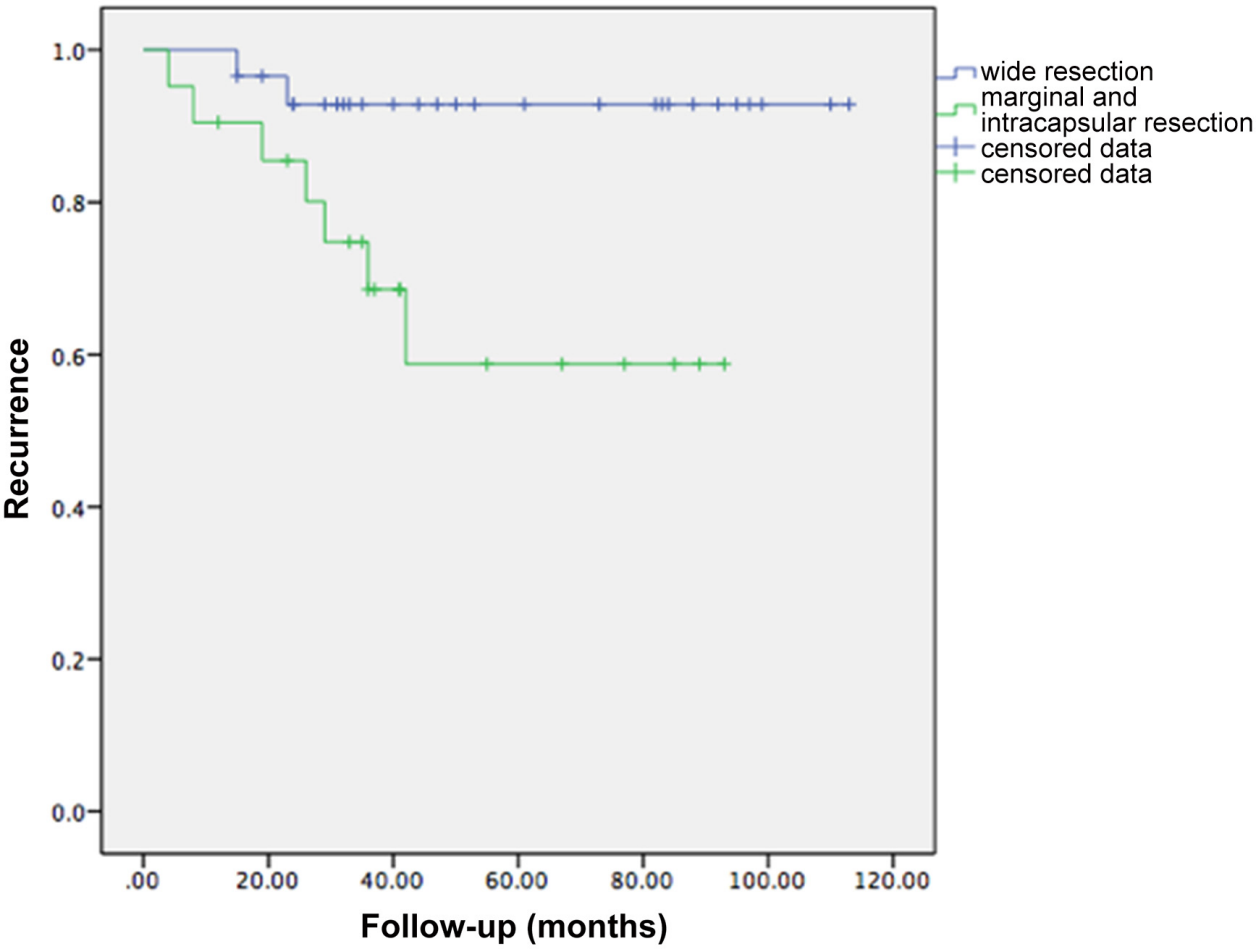
A Kaplan-Meier survival curve showing significant difference between recurrence rates of tumors with wide surgical margin and those with marginal or intracapsular margins (p = 0.02).

Thirty-two patients were alive at the most recent follow-up, with an estimated three-year and five-year survival rate of 66.3% and 57.5% according to the Kaplan-Meier survival analysis. Eighteen patients with 16 primary tumors and 2 metastatic malignancies died (from their diseases) an average of 28 months after surgery. Nine patients experienced local recurrence; the average disease-free interval for these patients was 23.6 months (range, 4–42 months) with an estimated one-year and three-year recurrence rate of 12.6% and 21.4% according to the Kaplan-Meier survival analysis. The recurrence rate in the patients with marginal or intralesional surgical margins was significantly greater than in those with wide margins (p = 0.02) (Fig 2). Of these recurrent cases, 1 patient with Ewing’s sarcoma received radiotherapy, 1 patient with osteosarcoma received second limb-salvage surgery, and 7 patients underwent hemipelvectomy. Twelve patients developed postoperative distant metastases at an average of 16 months (range, 4–40 months), and 11 of these patients died an average of 9.4 months after surgery (Table 3). The estimated one-year and three-year metastasis rate was 18.3% and 26.3%.

**Table 3:** Results of oncology and functional status, and complication analysis.

Study(year)	Cases	Follow-up (month)	Survival Rate	Recurrence Rate	MSTS Score	WP	DI	DL	PB	AL
**Current Study(2013)**	**50**	**54(12–113)**	**64%**	**18%**	**61.4%**	**28.0**	**14.0**	**4.0**	**10.0**	**2.0**
**T.Ji[15](2012)**	**100**	**53(24–103)**	**64%**	**20%**	**57.2%**	**18.0**	**15.0**	**9.0**	**5.0**	**2.0**
**Witte[16] (2009)**	**40**	**24(1–60)**	**82%**	**18%**	**50%**	**42.5**	**7.5**	**2.5**	**5.0**	**15.0**
**Jaiswal[18] (2008)**	**98**	**65(2–402)**	**68%**	**31%**	**59.4%**	**12**	**18.0**	**20.0**	**-**	**13.7**
**Tunn[19] (2007)**	**24**	**98**	**33%**	**20.8%**	**40% good**	**-**	**41.7**	**-**	**-**	**16.7**
**Ozaki[3] (2002)**	**12**	**57(26–77)**	**75%**	**33%**	**37%**	**-**	**25.0**	**8.3**	**-**	**25.0**
**Muller[2] (2002)**	**9**	**62(40–102)**	**67%**	**0%**	**11% good**	**22.2**	**55.6**	**11.0**	**-**	**22.2**

WP: Wound Problem, DI: Deep Infection, DL: Dislocation, PB: Prosthesis Breakage, AL: Aseptic Loosening

**Fig 3:**
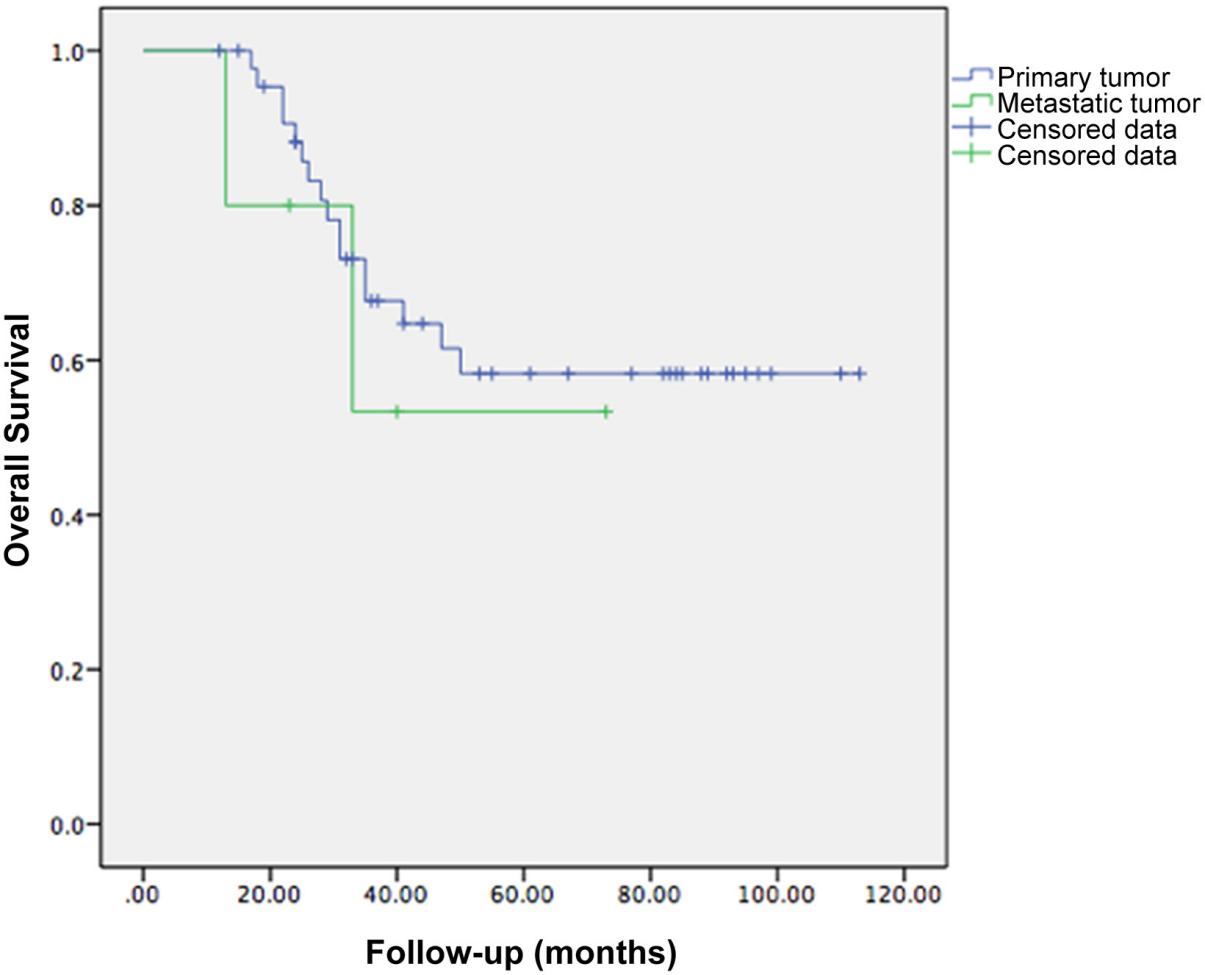
A Kaplan-Meier survival curve showing no significant difference between overall survival of primary and metastatic tumors (p = 0.60).

For the five patients of metastatic malignancies, three of them were alive at the most recent follow-up, two patients experienced local recurrence and died of the disease an average of 20 months after surgery. The survival rates (p = 0.60) and recurrence rates (p = 0.22) of metastatic malignancies did not differ significantly from that of primary tumors (Fig 3).

### Perioperative Complications

The average operative time was 6.8 hours (range, 4–13 hours), while the mean blood loss was 4200 ml (range, 600–20000 ml). Thirteen patients (26.0%) required transfer to the surgical intensive care unit (SICU) for 1 to 2 days due to unstable vital signs during surgery.

Fourteen patients (28%) had wound healing disturbances, including 5 cases of fat necrosis, 4 instances of skin necrosis and 5 superficial wound infections. Nine of these patients received a total of 11 debridement surgeries, and 4 patients also required vastus lateralis muscle flap transfer.

Seven patients (14%) developed deep infections, and the most common manifestations were mild fever, recurrent discharge or fistula. A total of 10 debridement surgeries were performed, combined with the intravenous administration of powerful antibiotics. Two patients required prosthesis removal without further reconstruction. Both patients underwent hemipelvectomy due to uncontrolled infection.

Other complications included one urethral injury, one sciatic nerve injury, and two deep vein thromboses(DVT). One patient of DVT progressed to pulmonary artery thromboembolism complicated by severe pneumonia and respiratory failure and was transferred to SICU. All other patients recovered after conservative therapy.

### Prosthesis-related Complications

**Fig 4:**
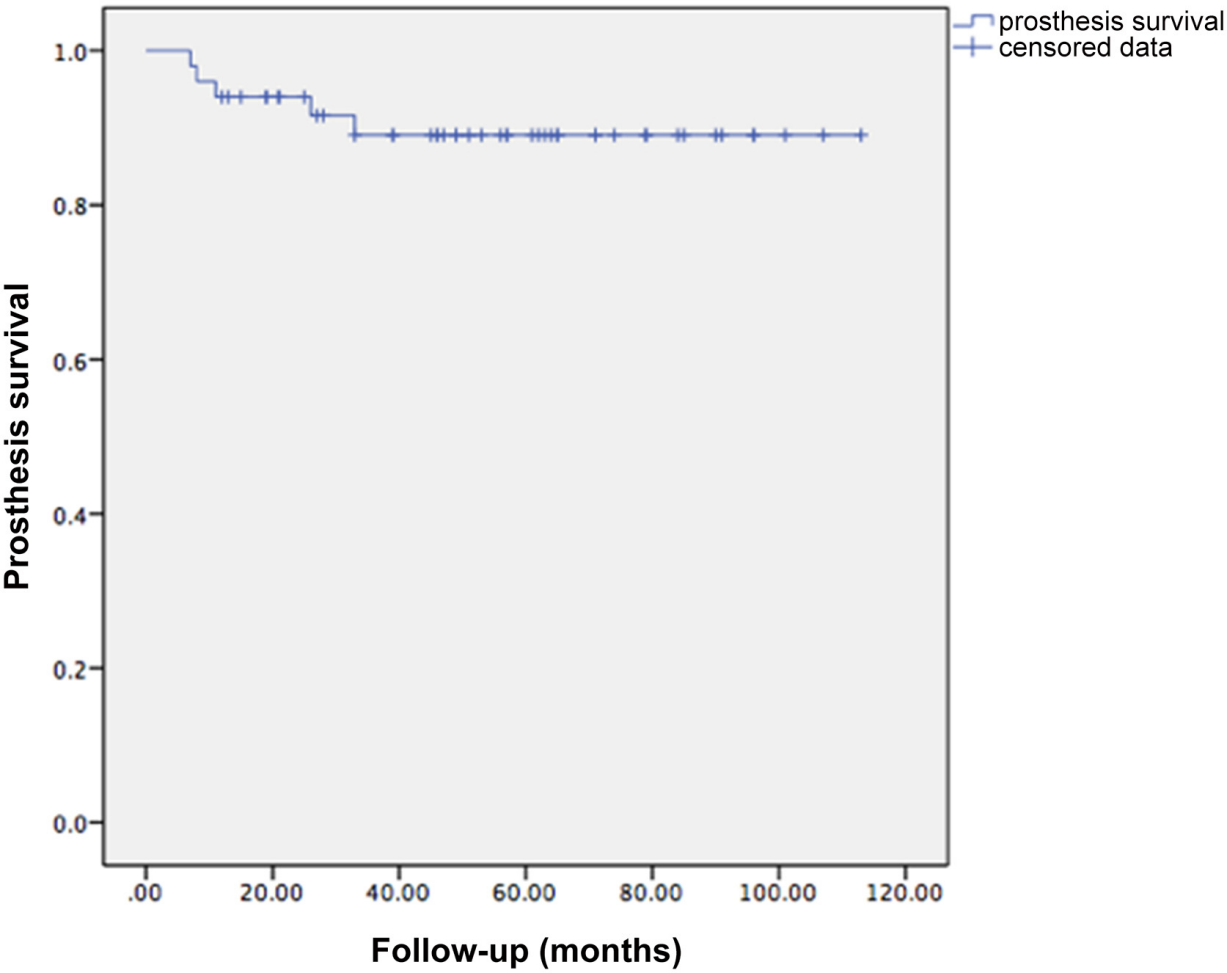
Prosthesis survival. A Kaplan-Meier survival curve showing prosthesis survival.

The total prosthesis-related complication rate was 16.0%. Five breakages at the pubic connection hardware, 1 aseptic loosening at the sacral connection and 2 dislocations of hip joint were observed (Table 3). Manipulative reduction was performed for the hip dislocations, while open reduction was necessary in one case. There were 2 septic and 3 aseptic implant failures, resulting in an overall 10% explantation rate (5 of 50 patients). Kaplan-Meier survival analysis was performed for prosthesis revision for any reason (Fig 4). The estimated three-year prosthesis survival rate was 89.0%.

### Functional Status

**Fig 5:**
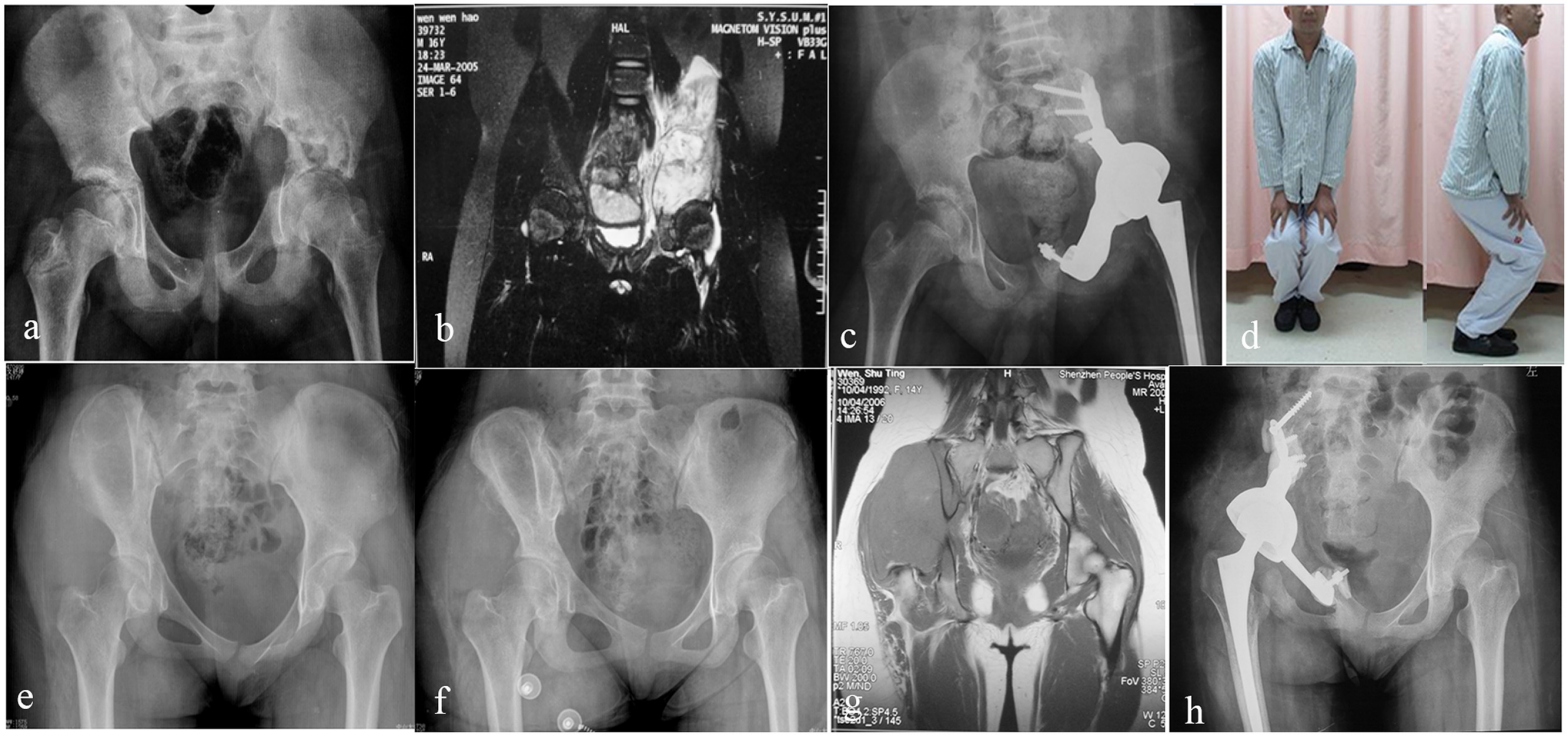
Patients follow-up. (a) and (b) Preoperative X-ray film and MRI of a patient with osteosarcoma involving section I/II of the left hemipelvis. (c) Postoperative X-ray film showing reconstruction with the modular hemipelvic endoprosthesis. (d) The function status of the hip joint of the same patient after reconstruction showing favorable extent of hip flexion. (e) Preoperative X-ray film of a patient with osteosarcoma involving section I/II of the right hemipelvis. (f) X-ray film after neo-adjuvant chemotherapy showing significantly enhanced osteogenesis inside the tumor. (g) Preoperative MRI. (h) Postoperative X-ray film showing reconstruction with the modular hemipelvic endoprosthesis.

The mean MSTS score was 61.4% (range, 37%-86%) (Table 3). The best-achieved parameters were those of pain reduction and emotional acceptance. All patients had limited walking ability and restricted lower limb function. Either a brace or a crutch was needed (Fig 5).

## Discussion

Limb-salvage surgery, combined with chemotherapy and radiotherapy, has replaced traditional hemipelvectomy in treating pelvic tumors because of the similar survival and recurrence rates[12–14]. Greater improvement in quality of life, as well as less psychological trauma and physical disability makes the procedure favorable and acceptable. Reconstruction with a hemipelvic endoprosthesis is the most common choice due to its reliability and effectiveness. However, the high complication rate and insufficient follow-up data makes this non-biological reconstruction strategy controversial. In addition, periacetabular tumors with invasion into the sacroiliac joint (section I, II, IV) were not included in our study due to difficulties with prosthetic fixation and stabilization after resecting the sacral wing. Hence, we have designed a novel reconstruction prosthetic system for these patients. The preliminary clinical experience and biomechanical findings of that system will be discussed elsewhere.

### Oncological Outcomes

The estimated three-year and five-year overall survival rate was 66.3% and 57.5%, with an estimated one-year and three-year recurrence rate of 12.6% and 21.4%, which was similar to that previously reported by T. Ji [15] and Witte [16]. In terms of the oncological outcomes, it was thought that metastatic malignancies, which have a poor life expectancy, should be differentiated from primary tumors. In our study, however, the survival rates and recurrence rates did not differ significantly between the primary and the metastatic tumors, and similar results have been reported in other articles[16, 17]. Patient selection criteria, therefore, should take into account patients with pelvic metastasis, and we would recommend this surgical procedure mainly for patients who present with a solitary metastasis of an otherwise well-controlled tumor.

We observed an estimated one-year and three-year recurrence rate of 12.6% and 21.4%, which is similar to that reported in other studies. The recurrence rate of metastatic malignancies did not differ significantly from that of primary tumors. However, we did find a significant relationship between surgical margin and recurrence rate, which has been reported by other authors[16, 18]. The recurrence rate of patients with marginal and intralesional surgical margins was significantly greater than those with wide margins. Therefore, wide surgical margins should be achieved to reduce the risk of recurrence, and microwave ablation is also helpful as it prevents tumor contamination while reducing hemorrhage. Results of long-term follow up will be reported in the future.

### Function

Acceptable functional results, with a mean MSTS score of 63.0%, were achieved. Retrospective investigation of other literature showed similar results. Sacrifice of glutei and atrophy of quadriceps aggravate function of the lower limb. The concept of restoring axial mechanical transmission instead of the anatomical pelvic ring is accepted. Therefore, iliac wing was not reconstructed anatomically to allow a better soft tissue coverage and to reduce wound-related complications, although it may lead to a poor shape of pelvis. The rotation center of the hip joint is shifted superomedially to shorten the gravity arm and to reduce the size of dead space. Theoretically, this process may aggravate gluteus weakness and limb shortening, but functional outcomes in our study showed no significant difference compared with other reports.

### Complications

Hemipelvic endoprosthetic reconstruction has not gained wide acceptance due to a relatively high complication rate. In our study, the overall complication rate reached 48.0%, and the most common complications were wound-related. Wound healing disturbances, which included fat necrosis, skin necrosis and superficial wound infection, resulted from large incisions, extensive soft tissue dissection and poor general condition. Delayed healing could be improved by intensive dressing changes combined with early debridement.

Deep infection is the most dangerous complication of surgeries with mega-implants. In our study, it occurred in 14.0% of the studied patients, which is lower compared with previously reported studies. Long surgical times, large dead cavity, poor soft tissue coverage and immunosuppressed conditions caused by neoadjuvant chemotherapy make deep infection difficult to control. High doses of sensitive intravenous antibiotics combined with debridement were used to treat deep infection. Although prosthesis removal without necessary further reconstruction was required in two patients, hemipelvectomy was performed due to uncontrolled infection. A superomedial shift of the hip center, combined with iliac wing depletion and vancomycin-containing cements, could decrease deep infection by reducing dead space and improving soft tissue coverage, but this solution still must be proven in long-term follow-up.

We observed a 16.0% prosthesis-related complication rate. Breakage at the pubic connection plate was the most common complication. It is not necessary, however, to restore the anterior pelvic girdle after breakage of the pubic connection plate, as previously reported by Guo[1]. In our study, only 1 of 5 patients with pubic connection plate breakage had revision surgery due to chronic inguinal pain. Revision surgery was performed only when instability occurred. A 3-year prosthesis survival rate of 89.0% was better achieved compared to recent reports[1, 16], disease recurrence was discussed alone and was excluded from implant failure for a better comparison. The overall complication rate was much higher than that of limb salvage surgery of the extremities, which could be explained by high complexity and difficulty of both the pelvic anatomy and surgical skills. Our results showed a higher estimated prosthetic survival rate, and the rates of dislocation and aseptic loosening were relatively lower than those previously reported, which could be attributed to the hip center shift and the "sacral hook," which transformed the great shearing force caused by the large angle between the sacral wing and the sacral connection component into compression force. Relevant biomechanical studies will be performed in the future to improve prosthetic design.

This study included patients for a large period, to investigate the impact of time on patient outcomes, 50 patients were divided into two groups by operation date of every five years. The result showed that oncology outcomes such as estimated survival, recurrence and metastasis were not improved significantly in this decade. This could be explained partially by the stagnation of adjuvant therapies and surgical skills. However, a significant reduction of blood loss and improvement of limb function have been observed, which were achieved by the use of microwave ablation and better rehabilitation. With a different follow-up period, the results of intragroup comparison need to be verified through long-term follow-up.

The shortcomings of this study included (1) a relatively short follow-up period for the recent patients. However, data of a minimum follow-up of 12 months was sufficient for investigating perioperative complications and assessing limb function, and had certain significance in analyzing oncology outcome since tumor metastasis and recurrence occurred mostly within two years postoperatively. (2) Patients were classified by location, and heterogeneity could be observed in diseases and type of resection, making it difficult to compare within group.

## Conclusions

Reconstruction with a modular hemipelvic endoprosthesis is reliable and effective, with improved function and an acceptable complication rate. Wide surgical margins should be achieved whenever possible.

## References

[1] Guo W, Li D, Tang X, Yang Y, Ji T. Reconstruction with modular hemipelvic prostheses for periacetabular tumor. Clin Orthop Relat Res. 2007;461:180–8. 10.1097/BLO.0b013e31806165d5 . 17452921

[2] Muller PE, Durr HR, Wegener B, Pellengahr C, Refior HJ, Jansson V. Internal hemipelvectomy and reconstruction with a megaprosthesis. International orthopaedics. 2002;26(2):76–9. . 12078881 10.1007/s00264-001-0322-4 PMC3620871

[3] Ozaki T, Hoffmann C, Hillmann A, Gosheger G, Lindner N, Winkelmann W. Implantation of hemipelvic prosthesis after resection of sarcoma. Clinical orthopaedics and related research. 2002;(396):197–205. . 11859244 10.1097/00003086-200203000-00030

[4] Falkinstein Y, Ahlmann ER, Menendez LR. Reconstruction of type II pelvic resection with a new peri-acetabular reconstruction endoprosthesis. The Journal of bone and joint surgery British volume. 2008;90(3):371–6. 10.1302/0301-620X.90B3.20144 . 18310763

[5] Langlais F, Lambotte JC, Thomazeau H. Long-term results of hemipelvis reconstruction with allografts. Clin Orthop Relat Res. 2001;(388):178–86. . 11451117 10.1097/00003086-200107000-00025

[6] Harrington KD. The use of hemipelvic allografts or autoclaved grafts for reconstruction after wide resections of malignant tumors of the pelvis. The Journal of bone and joint surgery American volume. 1992;74(3):331–41. . 1548259

[7] Ayvaz M, Bekmez S, Mermerkaya MU, Caglar O, Acaroglu E, Tokgozoglu AM. Long-term results of reconstruction with pelvic allografts after wide resection of pelvic sarcomas. ScientificWorldJournal. 2014;2014:605019 10.1155/2014/605019 24616637 PMC3925599

[8] Enneking WF. A system of staging musculoskeletal neoplasms. Clinical orthopaedics and related research. 1986;(204):9–24. . 3456859

[9] Enneking WF, Dunham WK. Resection and reconstruction for primary neoplasms involving the innominate bone. The Journal of bone and joint surgery American volume. 1978;60(6):731–46. . 701308

[10] Enneking W, Dunham W, Gebhardt M, Malawar M, Pritchard D. A system for the classification of skeletal resections. La Chirurgia degli organi di movimento. 1990;75(1 Suppl):217–40. . 2249538

[11] Enneking WF, Dunham W, Gebhardt MC, Malawar M, Pritchard DJ. A system for the functional evaluation of reconstructive procedures after surgical treatment of tumors of the musculoskeletal system. Clinical orthopaedics and related research. 1993;(286):241–6. . 8425352

[12] Ham SJ, Schraffordt Koops H, Veth RP, van Horn JR, Eisma WH, Hoekstra HJ. External and internal hemipelvectomy for sarcomas of the pelvic girdle: consequences of limb-salvage treatment. European journal of surgical oncology: the journal of the European Society of Surgical Oncology and the British Association of Surgical Oncology. 1997;23(6):540–6. . 9484927 10.1016/s0748-7983(97)93173-5

[13] Refaat Y, Gunnoe J, Hornicek FJ, Mankin HJ. Comparison of quality of life after amputation or limb salvage. Clin Orthop Relat Res. 2002;(397):298–305. . 11953621 10.1097/00003086-200204000-00034

[14] Mankin HJ, Hornicek FJ. Internal hemipelvectomy for the management of pelvic sarcomas. Surg Oncol Clin N Am. 2005;14(2):381–96. 10.1016/j.soc.2004.11.010 . 15817245

[15] Ji T, Guo W, Yang RL, Tang XD, Wang YF. Modular hemipelvic endoprosthesis reconstruction—experience in 100 patients with mid-term follow-up results. European journal of surgical oncology: the journal of the European Society of Surgical Oncology and the British Association of Surgical Oncology. 2013;39(1):53–60. 10.1016/j.ejso.2012.10.002 . 23131428

[16] Witte D, Bernd L, Bruns J, Gosheger G, Hardes J, Hartwig E, et al Limb-salvage reconstruction with MUTARS hemipelvic endoprosthesis: a prospective multicenter study. Eur J Surg Oncol. 2009;35(12):1318–25. 10.1016/j.ejso.2009.04.011 . 19477098

[17] Bruns J, Luessenhop SL, Dahmen G Sr. Internal hemipelvectomy and endoprosthetic pelvic replacement: long-term follow-up results. Archives of orthopaedic and trauma surgery. 1997;116(1–2):27–31. . 9006761 10.1007/BF00434096

[18] Jaiswal PK, Aston WJ, Grimer RJ, Abudu A, Carter S, Blunn G, et al Peri-acetabular resection and endoprosthetic reconstruction for tumours of the acetabulum. J Bone Joint Surg Br. 2008;90(9):1222–7. 10.1302/0301-620X.90B9.20758 . 18757964

[19] Tunn PU, Fehlberg S, Andreou D, Kettelhack C. [Endoprosthesis in the operative treatment of bone tumours of the pelvis]. Zeitschrift fur Orthopadie und Unfallchirurgie. 2007;145(6):753–9. 10.1055/s-2007-965757 . 18072042

